# GOAnnotator: linking protein GO annotations to evidence text

**DOI:** 10.1186/1747-5333-1-19

**Published:** 2006-12-20

**Authors:** Francisco M Couto, Mário J Silva, Vivian Lee, Emily Dimmer, Evelyn Camon, Rolf Apweiler, Harald Kirsch, Dietrich Rebholz-Schuhmann

**Affiliations:** 1 Departamento de Informática, Faculdade de Ciências, Universidade de Lisboa, Portugal; 2 European Bioinformatics Institute, Hinxton, Cambridge, UK

## Abstract

**Background:**

Annotation of proteins with gene ontology (GO) terms is ongoing work and a complex task. Manual GO annotation is precise and precious, but it is time-consuming. Therefore, instead of curated annotations most of the proteins come with uncurated annotations, which have been generated automatically. Text-mining systems that use literature for automatic annotation have been proposed but they do not satisfy the high quality expectations of curators.

**Results:**

In this paper we describe an approach that links uncurated annotations to text extracted from literature. The selection of the text is based on the similarity of the text to the term from the uncurated annotation. Besides substantiating the uncurated annotations, the extracted texts also lead to novel annotations. In addition, the approach uses the GO hierarchy to achieve high precision. Our approach is integrated into GOAnnotator, a tool that assists the curation process for GO annotation of UniProt proteins.

**Conclusion:**

The GO curators assessed GOAnnotator with a set of 66 distinct UniProt/SwissProt proteins with uncurated annotations. GOAnnotator provided correct evidence text at 93% precision. This high precision results from using the GO hierarchy to only select GO terms similar to GO terms from uncurated annotations in GOA. Our approach is the first one to achieve high precision, which is crucial for the efficient support of GO curators. GOAnnotator was implemented as a web tool that is freely available at .

## Background

The core objective of GOA (GO Annotation) is to provide high-quality GO (Gene Ontology) annotations to proteins within the UniProt Knowledgebase [1-3]. Manual GO annotation produces high-quality and granular GO term assignments, but tends to be slow and therefore covers less than 3% of UniProt. For better coverage, the GOA team integrates uncurated GO annotations deduced from automatic mappings between UniProt and other manually curated databases (e.g. Enzyme Commission numbers or InterPro domains). Although these assignments have high accuracy, the GOA team still has to verify them by extracting experimental results from peer-reviewed papers.

Reading these papers takes time, which motivates the research of text-mining methods. Very early on the text-mining system AbXtract was developed to identify keywords from MEDLINE abstracts and to score their relevance for a protein family [4]. Other systems have been developed in recent years to identify GO terms from the text: MeKE identified potential GO terms based on sequence alignment [5] and BioIE uses syntactic dependencies to select GO terms from the text [6]. Furthermore, other approaches use IT solutions where GO terminology is applied as a dictionary [7-10]. However, none of these systems have been integrated into the GOA curation process. Moreover, only Perez et al. makes use of the topology of the hierarchical structure of GO to measure the distance between two terms based on the number of edges that separate them. Neglecting the semantics of the hierarchical structure of GO causes incorrect annotations by over-predicting too deep-level GO terms, or useless annotations by predicting too general GO terms.

The selection of pieces of text that mention a GO term was assessed as part of the BioCreAtIvE competition [11]. This competition enabled the assessment of different text mining approaches and their ability to assist curators. The system with the best precision predicted 41 annotations, but 27 were not correct, which lead to a 35% precision (14 out of 41) [12]. The main problem is that GO was not designed for text mining. Its vocabulary is most of the times ambiguous and could not be easily deciphered by automatic processing and sometimes even by humans [13]. Without improvements to the precision, such automatic extractions are unhelpful to curators. This reflects the importance of designing more efficient tools to aid in the curation effort.

When manually annotating, GOA curators use pre-existing uncurated annotations as a guide, which can also be used to direct text-mining tools. Since GOA curators primarily require high precision in a text-mining solution, we expect that the information from the uncurated annotations will support this goal without the complex issues of creating rules and patterns encompassing all possible cases, and creating training sets that are too specific to be extended to new domains [14].

## Implementation

GOAnnotator is a tool for assisting the GO annotation of UniProt entries by linking the GO terms present in the uncurated annotations with evidence text automatically extracted from the documents linked to UniProt entries. Initially, the curator provides a UniProt accession number to GOAnnotator.

**Figure 1:**
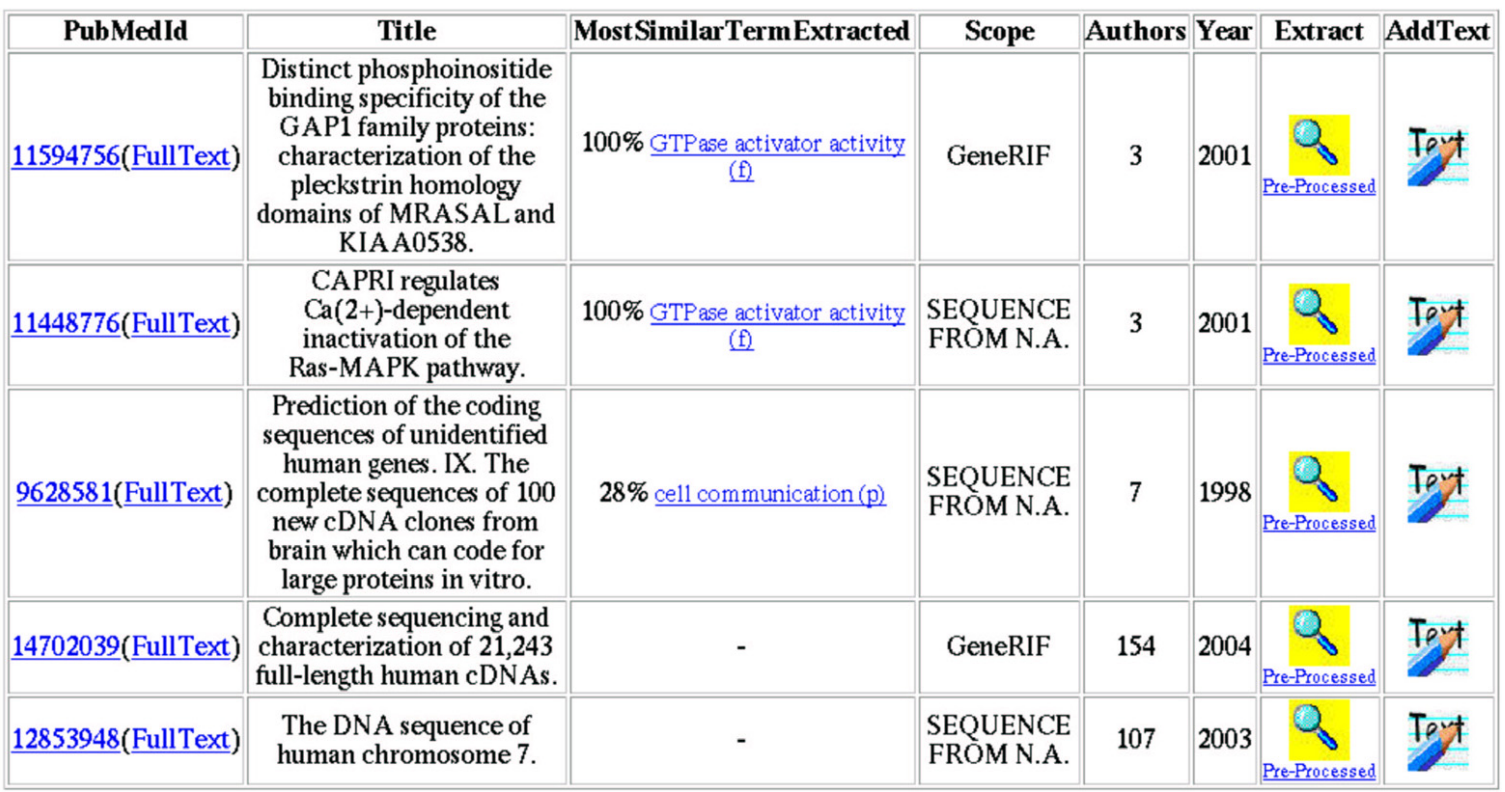
**List of documents related with a given protein**. The list is sorted by the most similar term extracted from each document. The curator can use the *Extract *option to see the extracted terms together with the evidence text. By default GOAnnotator uses only the abstract, but the curator can use the *AddText *option to replace or insert text.

GOAnnotator follows the bibliographic links found in the UniProt database and retrieves the documents. Additional documents are retrieved from the GeneRIF database or curators can add any other text [15]. GOAnnotator prioritizes the documents according to the extracted GO terms from the text and their similarity to the GO terms present in the protein uncurated annotations (see Figure 1). Any extracted GO term is an indication for the topic of the document, which is also taken from the UniProt entry. The curator uses the topic as a hint to potential GO annotation.

The extraction of GO terms is based on FiGO, a method used for the BioCreAtIvE competition [16]. FiGO receives a piece of text and returns the GO terms that were detected in the given text. To each GO term, FiGO assigns a confidence value that represents the terms' likelihood of being mentioned in the text. The confidence value is the ratio of two parameters. The first parameter is called local evidence context and is used to measure the likelihood that words in the text are part of a given GO term. The second parameter is a correction parameter, which increases the confidence value when the words detected in the text are infrequent in GO. In BioCreAtIvE, FiGO predicted 673 annotations but 615 were not correct, which lead to a 8.6% precision (58 of 673).

GO terms are considered to be similar if they are in the same lineage or if they share a common parent in the GO hierarchy. To calculate a similarity value between two GO terms, we decided to implement a semantic similarity measure. Research on Information Theory proposed many semantic similarity measures. Some of them calculate maximum likelihood estimates for each concept using the corpora, and then calculate the similarity between probability distributions. Semantic similarity measures combine the structure of an ontology with their information content based on statistical data from corpora [17]. The information content of a concept is inversely proportional to its frequency in the corpora. Concepts that are frequent in the corpora have low information content. In case of GO the corpora used to derive the statistical information is the annotations provided by GO, i.e. the information content of a GO term is calculated based on the number of proteins annotated to it. For example, GO terms annotated to most of the proteins normally provide little semantic information.

Many semantic similarity measures applied to ontologies have been developed. We implemented a measure based on the ratio between the information content of the most informative common ancestor and the information content of both concepts [18]. Recent studies studied the the effectiveness of semantic similarity measures over the GO [19,20]. The results showed that GO similarity is correlated with sequence and family similarity, i.e., they demonstrated the feasibility of using semantic similarity measures in a biological setting.

**Figure 2:**
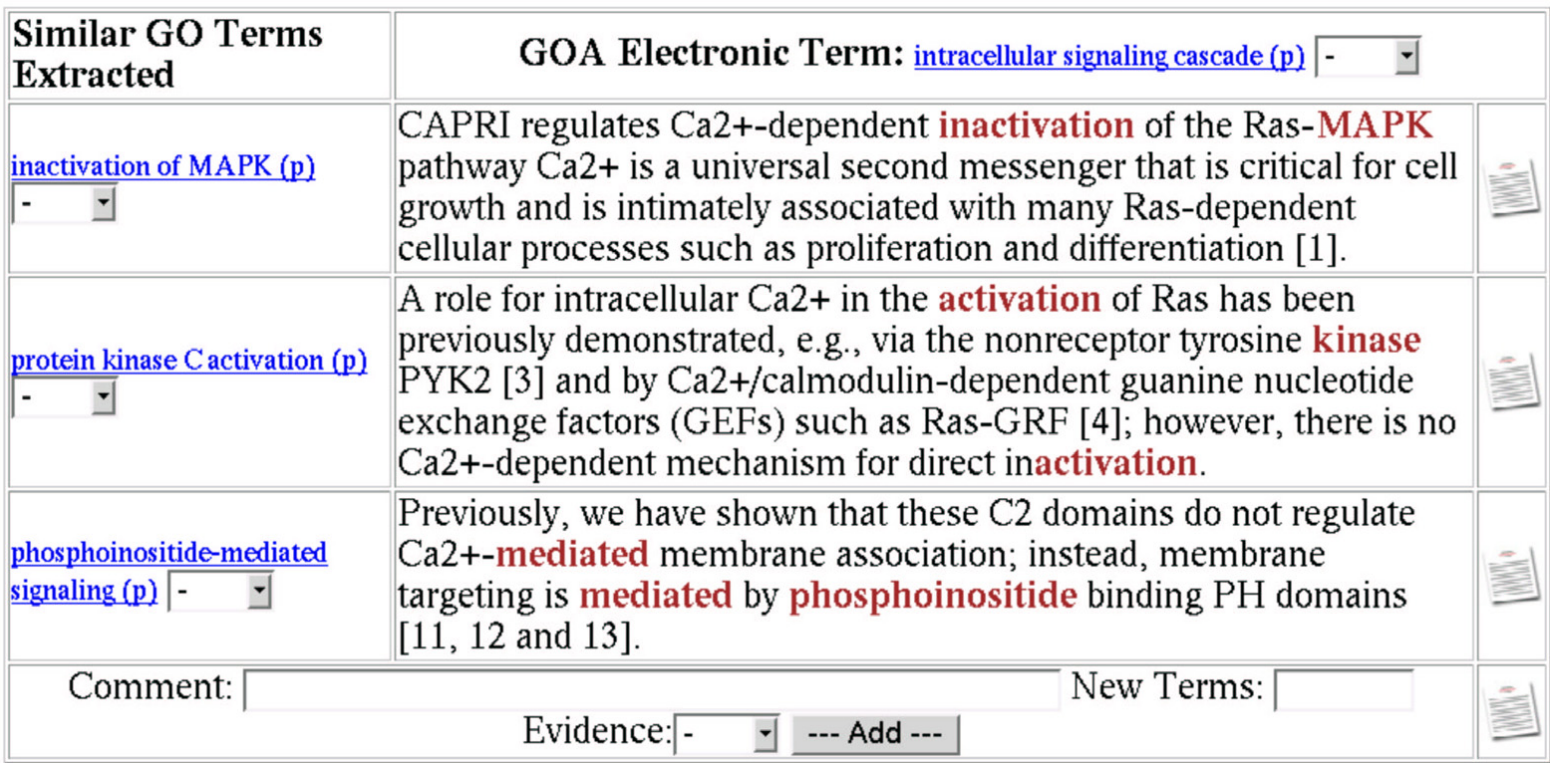
**GO terms extracted**. For each uncurated annotation, GOAnnotator shows the similar GO terms extracted from a sentence of the selected document. If any of the sentences provides correct evidence for the uncurated annotation, or if the evidence supports a GO term similar to that present in the uncurated annotation, the curator can use the *Add *option to store the annotation together with the document reference, the evidence codes and any comments.

GOAnnotator displays a table for each uncurated annotation with the GO terms that were extracted from a document and were similar to the GO term present in the uncurated annotation (see Figure 2). The sentences from which the GO terms were extracted are also displayed. Words that have contributed to the extraction of the GO terms are highlighted. GOAnnotator gives the curators the opportunity to manipulate the confidence and similarity thresholds to modify the number of predictions.

## Results

The GOA team has volunteered to curate about 3% proteins of a list of 1953 uncurated UniProt/SwissProt proteins. Thus, we decreased the similarity and confidence thresholds of GOAnnotator until we get this percentage. We stopped at a 40% similarity and a 50% confidence thresholds, resulting in only 66 proteins. This means that GOAnnotator identified evidence texts with more than 40% similarity and 50% confidence for these 66 proteins. For 80 uncurated annotations to these proteins, GOAnnotator extracted 89 similar annotations and their evidence text from 118 MEDLINE abstracts. The 80 uncurated annotations included 78 terms from different domains of GO (see Table 1). After analyzing the 89 evidence texts, GOA curators found that 83 were valid to substantiate 77 distinct uncurated annotations (see Table 2), i.e. 93% precision. Table 3 shows that 78% (65 out of 83) of the correct evidence texts confirmed the uncurated annotations, i.e. the extracted annotation and the uncurated annotation contained the same GO identifier. In cases where the evidence text was correct, not always it contained exactly any of the known variations of the extracted GO term. In the other cases the extracted GO term was similar: in 15 cases the extracted GO term was in the same lineage of the GO term in the uncurated annotation; in 3 cases the extracted GO term was in a different lineage, but both terms were similar (share a parent). In general, we can expect GOAnnotator to confirm the uncurated annotation using the findings from the scientific literature, but it is obvious as well that GOAnnotator can propose new GO terms.

**Table 1:** Distribution of the GO terms from the selected uncurated annotations through the different aspects of GO.

GO Aspect	GO Terms
molecular function	54
biological process	18
cellular component	6
total	78

**Table 2:** Evaluation of the evidence text substantiating uncurated annotations provided by the GOAnnotator.

Evidence Evaluation	Extracted Annotations
correct	83
incorrect	6
total	89

**Table 3:** Comparison between the extracted GO terms with correct evidence text and the GO terms from the uncurated annotations.

GO Terms	Extracted Annotations
exact	65
same lineage	15
different lineage	3
total	83

### Examples

GOAnnotator provided correct evidence for the uncurated annotation of the protein "Human Complement factor B precursor" (P00751) with the term "complement activation, alternative pathway" (GO:0006957). The evidence is the following sentence from the document with the PubMed identifier 8225386: "The human complement factor B is a centrally important component of the alternative pathway activation of the complement system."

GOAnnotator provided a correct evidence for the uncurated annotation of the protein "U4/U6 small nuclear ribonucleoprotein Prp3" (O43395) with the term "nuclear mRNA splicing, via spliceosome" (GO:0000398). From the evidence the tool extracted the child term "regulation of nuclear mRNA splicing, via spliceosome" (GO:0048024). The evidence is the following sentence from the document with the PubMed identifier 9328476: "Nuclear RNA splicing occurs in an RNA-protein complex, termed the spliceosome." However, this sentence does not provide enough evidence on its own, the curator had to analyze other parts of the document to draw a conclusion.

GOAnnotator provided a correct evidence for the uncurated annotation of the protein "Agmatinase" (Q9BSE5) with the term "agmatinase activity" (GO:0008783). From the evidence the tool extracted the term "arginase activity" (GO:0004053) that shares a common parent. The evidence was provided by the following sentence from the document with the PubMed identifier 11804860: "Residues required for binding of Mn(2+) at the active site in bacterial agmatinase and other members of the arginase superfamily are fully conserved in human agmatinase." However, the annotation only received a NAS (Non-traceable author statement) evidence code, as the sentence does not provide direct experimental evidence of arginase activity. Papers containing direct experimental evidence for the function/subcellular location of a protein are more valuable to GO curators.

GOAnnotator provided a correct evidence for the uncurated annotation of the protein "3'–5' exonuclease ERI1" (Q8IV48) with the term "exonuclease activity" (GO:0004527). The evidence is the following sentence from the document with the PubMed identifier 14536070: "Using RNA affinity purification, we identified a second protein, designated 3'hExo, which contains a SAP and a 3' exonuclease domain and binds the same sequence." However, the term "exonuclease activity" is too high level, and a more precise annotation should be "3'–5' exonuclease activity" (GO:0008408).

## Discussion

Researchers need more than facts, they need the source from which the facts derive [21]. GOAnnotator provides not only facts but also their evidence, since it links existing annotations to scientific literature. GOAnnotator uses text-mining methods to extract GO terms from scientific papers and provides this information together with a GO term from an uncurated annotation. In general, we can expect GOAnnotator to confirm the uncurated annotation using the findings from the scientific literature, but it is obvious as well that GOAnnotator can propose new GO terms. In both cases, the curator profits from the integration of both approaches into a single interface. By comparing both results, the curator gets convenient support to take a decision for a curation item based on the evidence from the different data resources.

GOAnnotator provided correct evidence text at 93% precision, and in 78% of these cases the GO term present in the uncurated annotation was confirmed. These results were obtained for a small subset of the total number of uncurated annotations, but it represents already a significant set for curators. Notice that manual GO annotation covers less than 3% of UniProt. Over time, proteins tend to be annotated with more accurate uncurated terms and bibliography. Thus, the percentage of uncurated proteins satisfying the 40% similarity and 50% confidence thresholds will grow, and therefore make GOAnnotator even more effective. Sometimes, the displayed sentence from the abstract of a document did not contain enough information for the curators to evaluate an evidence text with sufficient confidence. Apart from the association between a protein and a GO term, the curator needs additional information, such as the type of experiments performed and the species from which the protein originates. Unfortunately, quite often this information is only available in the full text of the scientific publication. GOAnnotator can automatically retrieve the abstracts, but in the case of the full text the curator has to copy and paste the text into the GOAnnotator interface, which only works for a limited number of documents. BioRAT solve this problem by retrieving full text documents from the Internet [22]. In addition, the list of documents cited in the UniProt database was not sufficient for the curation process. In most cases, the curators found additional sources of information in PubMed. In the future, GOAnnotator should be able to automatically query PubMed using the protein's names to provide a more complete list of documents.

GOAnnotator ensures high accuracy, since all GO terms that did not have similar GO terms in the uncurated annotations were rejected. Using this 40% similarity threshold may filter out meaningful potential annotations that are not similar to known curated annotations. However, without this restriction the results returned by the text mining method would contain too much noise to be of any use to curators, as it was demonstrated in the BioCreAtIvE competition. GOAnnotator meets the GOA team's need for tools with high precision in preference to those with high recall, and explains the strong restriction for the similarity of two GO terms: only those that were from the same lineage or had a shared parent were accepted. Thus, GOAnnotator not only predicted the exact uncurated annotation but also more specific GO annotations, which was of strong interest to the curators. MeKE selected a significant number of general terms from the GO hierarchy [5]. Others distinguished between gene and family names to deal with general terms [7]. GOAnnotator takes advantage of uncurated annotations to avoid general terms by extracting only similar terms, i.e. popular proteins tend to be annotated to specific terms and therefore GOAnnotator will also extract specific annotations to them.

The applied text-mining method FiGO was designed for recognizing terms and not for extracting annotations, i.e. sometimes the GO term is correctly extracted but is irrelevant to the actual protein of interest. The method also generated mispredictions in the instances where all the words of a GO term appeared in disparate locations of a sentence or in an unfortunate order. Improvements can result from the incorporation of better syntactical analysis into the identification of GO terms similar to the techniques used by BioIE [6]. For example, a reduction of the window size of FiGO or the identification of noun phrases can further increase precision. In the future, GOAnnotator can also use other type of text-mining methods that prove to be more efficient for extracting annotations.

## Conclusion

We presented GOAnnotator, a system that automatically identifies evidence text in literature for GO annotation of Uniprot/SwissProt proteins. GOAnnotator provided evidence text at high precision (93%, 66 sample proteins) taking advantage of existing uncurated annotations and the GO hierarchy. GOAnnotator incorporates a text-mining method to extract GO terms from text, and a similarity measure to select GO terms similar to GO terms from uncurated annotations.

GOAnnotator assists the curation process by allowing fast verification of uncurated annotations from evidence texts, which can also be the source for novel annotations. GOAnnotator is available through a Web interface, which enables the verification of uncurated annotations of any UniProt entry with evidence extracted from literature.

## Availability and requirements

**Project name: **ReBIL – Relating Biological Information through Literature

**Project home page: **

**System home page: **

**Operating system: **Linux on server side, platform independent on client sides

**Programming language: **Java and PHP on the server side

**Other requirements: **Available from any Internet browser on the client side

**License: **Free access

**Any restrictions to use by non-academics: **No restrictions

## Competing interests

The author(s) declare that they have no competing interests.

## Authors' contributions

FC carried out the system development and drafted the manuscript. MS and RA supported the study, and helped to draft the manuscript. VL, ED and EC participated in the design of the system and performed its assessment. HK and DR participated in its design and coordination and helped to draft the manuscript. All authors collaborated since the beginning of the project. All authors read and approved the final manuscript.

## References

[B1] Camon E, Magrane M, Barrell D, Lee V, Dimmer E, Maslen J, Binns D, Harte N, Lopez R, Apweiler R (2004). The Gene Ontology Annotations (GOA) Database: sharing knowledge in UniProt with Gene Ontology. Nucleic Acids Research.

[B2] GO-Consortium (2004). The Gene Ontology (GO) database and informatics resource. Nucleic Acids Research.

[B3] Apweiler R, Bairoch A, Wu C, Barker W, Boeckmann B, Ferro S, Gasteiger E, Huang H, Lopez R, Magrane M, Martin M, Natale D, O'Donovan C, Redaschi N, Yeh L (2004). UniProt: the Universal Protein Knowledgebase. Nucleic Acids Research.

[B4] Andrade M, Valencia A (1998). Automatic Extraction of Keywords from Scientific Text: Application to the Knowledge Domain of Protein Families. Bioinformatics.

[B5] Chiang J, Yu H (2003). MeKE: discovering the functions of gene products from biomedical literature via sentence alignment. Bioinformatics.

[B6] Kim J, Park J (2004). BioIE: retargetable information extraction and ontological annotation of biological interactions from literature. Journal of Bioinformatics and Computational Biology.

[B7] Koike A, Niwa Y, Takagi T (2005). Automatic extraction of gene/protein biological functions from biomedical text. Bioinformatics.

[B8] Pérez A, Perez-Iratxeta C, Bork P, Thode G, Andrade M (2004). Gene annotation from scientific literature using mappings between keyword systems. Bioinformatics.

[B9] Müller H, Kenny E, Sternberg P (2004). Textpresso: an ontology-based information retrieval and extraction system for biological literature. PLOS Biology.

[B10] Rebholz-Schuhmann D, Kirsch H, Gaudan MAS, Rynbeek M, Stoehr P (2006). Protein annotation by EBIMed. Nature Biotechnology.

[B11] Hirschman L, Yeh A, Blaschke C, Valencia A (2005). Overview of BioCreAtIvE: critical assessment of information extraction for biology. BMC Bioinformatics.

[B12] Chiang J, Yu H (2004). Extracting Functional Annotations of Proteins Based on Hybrid Text Mining Approaches. Proc of the BioCreAtIvE Challenge Evaluation Workshop.

[B13] Camon E, Barrell D, Dimmer E, Lee V, Magrane M, Maslen J, Binns D, Apweiler R (2005). An evaluation of GO annotation retrieval for BioCreAtIvE and GOA. BMC Bioinformatics.

[B14] Couto F, Silva M Advanced Data Mining Techonologies in Bioinformatics, Idea Group Inc 2006 chap Mining the BioLiterature: towards automatic annotation of genes and proteins.

[B15] Mitchell J, Aronson A, Mork J, Folk L, Humphrey S, Ward J (2003). Gene indexing: characterization and analysis of NLM's GeneRIFs. Proc of the AMIA 2003 Annual Symposium.

[B16] Couto F, Silva M, Coutinho P (2005). Finding Genomic Ontology Terms in Text using Evidence Content. BMC Bioinformatics.

[B17] Rada R, Mili H, Bicknell E, Blettner M (1989). Development and application of a metric on semantic nets. IEEE Transactions on Systems.

[B18] Lin D (1998). An information-theoretic definition of similarity. Proc of the 15th International Conference on Machine Learning.

[B19] Lord P, Stevens R, Brass A, Goble C (2003). Investigating semantic similarity measures across the Gene Ontology: the relationship between sequence and annotation. Bioinformatics.

[B20] Couto F, Silva M, Coutinho P (2006). Measuring Semantic Similarity between Gene Ontology Terms. DKE – Data and Knowledge Engineering, Elsevier Science.

[B21] Rebholz-Schuhmann D, Kirsch H, Couto F (2005). Facts from text – Is Text Mining ready to deliver?. PLoS Biology.

[B22] Corney D, Buxton B, Langdon W, Jones D (2004). BioRAT: Extracting Biological Information from full-length papers. Bioinformatics.

